# Robot-Assisted Total Gastrectomy for Juvenile Polyposis Syndrome-Associated Gastric Cancer Performed in a Patient with Factor XIII Deficiency: A Case Report

**DOI:** 10.70352/scrj.cr.26-0053

**Published:** 2026-05-01

**Authors:** Toshifumi Saito, Toru Ishiguro, Hideyuki Ishida, Kurumi Kiuchi, Naoko Irie, Hiroyasu Ishikawa, Norimichi Chiyonobu, Tetsuya Ito, Yoshiko Mori, Youichi Kumagai, Noriko Tanabe

**Affiliations:** 1 Department of Digestive Tract and General Surgery, Saitama Medical Center, Saitama Medical University, Kawagoe, Saitama, Japan; 2 Department of Clinical Genetics, Saitama Medical Center, Saitama Medical University, Kawagoe, Saitama, Japan

**Keywords:** gastric cancer, factor XIII deficiency, juvenile polyposis syndrome

## Abstract

**INTRODUCTION:**

Factor XIII deficiency (FXIIID) is a rare coagulation disorder that can cause severe or delayed bleeding and impair wound healing despite normal routine coagulation test results. Congenital FXIIID is caused by homozygous germline pathogenic variants of *F13A* or *F13B*. Herein, we report the successful treatment of a patient with FXIIID who underwent surgery for juvenile polyposis syndrome (JPS)-associated early gastric cancer.

**CASE PRESENTATION:**

A 44-year-old woman was admitted to the hospital with anemia secondary to menorrhagia. Gastrointestinal examination revealed polyposis throughout the stomach and type 0–Is early gastric cancer, which was consistent with the histological diagnosis of the biopsy specimens. Investigation into her family history revealed that her mother and maternal aunt had gastric polyposis. Preoperative laboratory tests were almost normal, except for low levels of hemoglobin (6.0 g/dL) and Factor XIII (FXIII) activity (31%). Robot-assisted total gastrectomy with D1-level lymph node dissection, followed by Roux-en-Y reconstruction, was successfully performed, with an operative time of 347 min and estimated blood loss of 26 mL. To prevent surgery-related bleeding, preoperative and postoperative administration of FXIII concentrate (30 IU/kg) was planned. The postoperative course was uneventful, and the patient was discharged on POD 9. Genetic testing identified a germline pathogenic frameshift variant in *SMAD4* (c.96delT [p.Ser32fs*13]), which was consistent with the phenotype of JPS, and a heterozygous missense variant of uncertain significance in *F13A1* (c.308A>T [p.Glu103Val]). Nine months postoperatively, FXIII activity was re-evaluated and remained low at 59%, although it was higher than the preoperative level.

**CONCLUSIONS:**

Recognition of FXIIID and appropriate perioperative management may help prevent unexpected bleeding and enable safe gastrectomy in patients with FXIIID.

## Abbreviations

aPTT
: activated partial thromboplastin time
CA125
: carbohydrate antigen 125
CA19-9
: carbohydrate antigen 19-9
CEA
: carcinoembryonic antigen
FXIII
: factor XIII
FXIIID
: factor XIII deficiency
JPS
: juvenile polyposis syndrome
PT
: prothrombin time

## INTRODUCTION

FXIII plays a crucial role in the maintenance and stabilization of fibrin clots in the coagulation cascade. FXIIID can lead to a bleeding tendency and may cause massive hemorrhage during childbirth or surgical procedures.^1,2)^ The causes of FXIIID include congenital and acquired forms. Congenital FXIIID is an autosomal recessive disease caused by biallelic germline pathogenic variants of *F13A1* or *F13B*.^2,3)^ The prevalence of congenital FXIIID is estimated to be 1 in 2–3 million live births.^2)^ Severe congenital FXIIID often manifests in the neonatal period, with serious bleeding symptoms, such as umbilical stump bleeding and cephalohematoma, occurring in approximately 80% of affected newborns. Intracranial hemorrhage, which represents the most common cause of death in patients with FXIIID, has been reported in nearly 30% of neonates with severe congenital FXIIID.^3)^ In addition, previous studies have reported that patients with FXIIID, particularly females and older individuals, may present with a wide range of bleeding manifestations, including recurrent spontaneous abortions, impaired wound healing, menorrhagia, frequent nose bleeds, hematuria, and easy bruising.^3)^ In recent years, the concept of mild congenital FXIIID has been proposed.^4–6)^ Mild congenital FXIIID is associated with heterozygous and hemiallelic variants of *F13A1* or *F13B*. Patients with mild congenital FXIIID are usually asymptomatic; however, bleeding tendency may become apparent when individuals are exposed to physical stress such as trauma, childbirth, or surgical intervention.^6)^ In contrast, acquired FXIIID is associated with autoimmune, idiopathic, and hyper-consumption–related etiologies.^2)^

In surgical settings, especially during major abdominal procedures, the perioperative management of FXIIID remains clinically challenging because of the risk of recurrent or delayed bleeding. Herein, we report an extremely rare case of a patient with FXIIID and JPS-associated early gastric cancer, who was successfully treated with total gastrectomy and planned administration of FXIII concentrate.

## CASE PRESENTATION

A 44-year-old woman was referred to another hospital complaining of dizziness. She had a history of menorrhagia, but no history of surgery or blood transfusion. Her family history included gastric polyposis in her mother and maternal aunt. Her older brother and her 3 children were healthy. She was admitted to the hospital for anemia secondary to menorrhagia. Blood tests performed at the referring hospital revealed severe anemia with a hemoglobin level of 5.0 g/dL. Despite normal routine coagulation tests, the menorrhagia and anemia were severe. Further evaluation for bleeding disorders revealed an FXIII activity of 28%. These findings led to a diagnosis of FXIII deficiency of unknown etiology. She received a transfusion of 4 units of packed red blood cells for the treatment of anemia. Endoscopic examinations were performed for melena, and upper gastrointestinal endoscopy revealed gastric polyposis and a type 0–Is tumor in the antrum. The patient was referred to our hospital for further treatment.

**Fig. 1:**
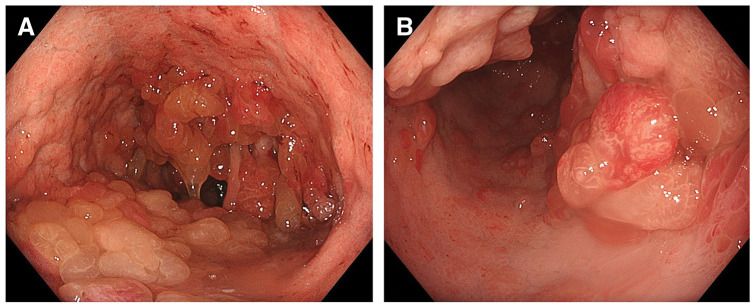
Endoscopic findings of the stomach. (**A**) Endoscopic image of the gastric body showing numerous cystic polyps with normal coloration densely distributed throughout the mucosa. Polyps were diffusely present from the cardia to the antrum. (**B**) Endoscopic image of the antrum. On the posterior wall, multiple polyps had coalesced, presenting a reddish appearance with vascular dilation and proliferation. Biopsy from this area revealed adenocarcinoma.

Hematological and biochemical examinations performed at our institution revealed persistent anemia, with a hemoglobin level of 6.0 g/dL. The platelet count, PT, and aPTT were all within normal ranges; however, FXIII activity remained decreased at 31%. The levels of tumor markers, including CEA, CA19-9, and CA125, were not elevated. Colonoscopy at our institution revealed a 40-mm type 0–Isp polyp in the descending colon, which was resected by endoscopic submucosal dissection. Histopathological examination of the resected specimen revealed tubulovillous adenoma with both low- and high-grade dysplasia. Upper gastrointestinal endoscopy revealed numerous edematous polyps throughout the stomach and a type 0–Is tumor in the antrum (**Fig. 1A** and **1B**). Biopsy revealed a hyperproliferative gastric epithelium, interstitial edema, and well-differentiated adenocarcinoma. Contrast-enhanced CT of the chest and abdomen showed an enhanced mass lesion in the antrum of the stomach, with no evidence of regional lymph node enlargement or distant metastasis.

Based on the family history, endoscopic findings, and histopathological features, the patient was diagnosed with JPS-associated early gastric cancer.

### Diagnosis and perioperative management

Gastric cancer was classified as L, Ant–Less, type 0–Is, cT1bN0M0, cStage I according to the 15th edition of the Japanese Classification of Gastric Carcinoma by the Japanese Gastric Cancer Association.^7)^ Four units of packed red blood cells were administered preoperatively for anemia. To prevent perioperative bleeding, FXIII concentrate (Fibrogammin-P; CSL Behring, King of Prussia, PA, USA) was administered at a dose of 30 IU/kg on the day before surgery. Robot-assisted total gastrectomy with D1 lymph node dissection and Roux-en-Y reconstruction was performed. Because the patient had FXIIID with a potential risk of perioperative bleeding, the extent of lymph node dissection was limited to D1, and a minimally invasive robotic approach was selected. The operative time was 347 min. No remarkable intraoperative bleeding was observed, and the estimated blood loss was 26 mL. An additional dose of FXIII concentrate (30 IU/kg) was administered to prevent postoperative bleeding. The patient’s postoperative course was uneventful, with no evidence of bleeding or other complications. The drain was removed on POD 7, and the patient was discharged on day 9. The patient did not experience menorrhagia after gastrectomy. Nine months after surgery, her hemoglobin level was 9.8 g/dL, and FXIII activity was 59%.

### Macroscopic and histopathological findings

**Fig. 2:**
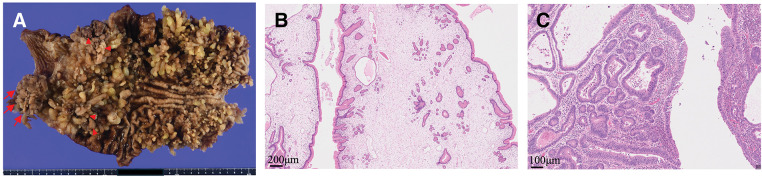
Macroscopic and histopathological findings of the resected specimen. (**A**) Macroscopic view showing diffuse gastric polyposis throughout the stomach. Arrows indicate the 35-mm adenocarcinoma, while arrowheads indicate additional intramucosal adenocarcinomas measuring <10 mm. (**B**) Low-power histological findings of multiple polyps showing prominent stromal edema, consistent with juvenile polyps. (**C**) Histopathological findings showing a tumor composed of well-formed tubular structures resembling normal glands, consistent with well-differentiated tubular adenocarcinoma.

Macroscopic findings of the resected specimen showed diffuse gastric polyposis throughout the stomach. A 35-mm tumor was identified on the posterior wall of the antrum (**Fig. 2A**). Histopathological examination revealed multiple juvenile polyps in the background mucosa (**Fig. 2B**). The tumor located in the antrum was a well-differentiated tubular adenocarcinoma (**Fig. 2C**) with the following characteristics: L, Ant, 35 × 25 × 20 mm, type 0–IIa, tub1, pT1a (M), ly0, v0, pPM0, pDM0, pN0, cN0, and pStage IA. Four additional intramucosal carcinomas (pT1a), each <10 mm in size, were identified. Multiple juvenile polyps were observed in the background mucosa.

### Genetic testing

The germline variants of *F13A1* and *F13B* associated with congenital FXIIID were examined using next-generation sequencing at the Laboratory of Genetic Diagnosis, Kazusa DNA Research Institute. No germline variants were detected in the *F13B* gene. A heterozygous missense variant of the *F13A1* gene (NM_000129.4), c.308A>T (p.Glu103Val), was identified. This variant was classified as a variant with uncertain significance. Furthermore, multigene panel testing to identify hereditary gastrointestinal polyposis syndromes and hereditary colorectal cancer syndromes, including *SMAD4* and *BMPR1A* genes associated with JPS, identified a germline pathogenic variant in *SMAD4* (NM_005359.6), c.96delT (p.Ser32fs*13), genetically confirming the phenotype of JPS.

### Management of the patient’s relatives

The patient’s older brother and 3 children were considered at risk of having JPS and/or FXIIID. Genetic testing, endoscopic examination, and FXIII measurements have been scheduled for these 4 individuals. Although endoscopic examinations have not yet been performed, blood testing revealed reduced FXIII activity (60%) in 1 of the patient’s 3 daughters, and she was scheduled for follow-up.

## DISCUSSION

Routine preoperative coagulation tests such as PT and aPTT are typically normal in FXIIID,^1,2)^ making the diagnosis challenging. FXIIID is defined as an FXIII activity of less than 70%.^1)^ In patients with FXIIID, failure of fibrin stabilization can lead to massive or delayed bleeding and impaired wound healing.^2)^ Congenital FXIIID has traditionally been considered an autosomal recessive disorder caused by germline pathogenic variants in *F13A1* or *F13B*. In contrast, acquired FXIIID caused by autoantibodies is even rarer than that caused by the congenital forms^8)^; fewer than 100 cases with autoantibodies against FXIII have been reported worldwide.^8,9)^ Acquired FXIIID is generally associated with severe bleeding and advanced age; thus, the clinical features of the present case were not fully consistent with an acquired etiology. The present patient was suspected to have congenital FXIIID rather than acquired FXIIID, although assessment of IgG-type autoantibodies against FXIII was unavailable at our institution.

In recent years, the concept of mild congenital FXIIID has been proposed.^3,6)^ Patients with this condition typically show plasma FXIII activity levels between 20% and 60% and are usually asymptomatic; however, bleeding manifestations may become apparent when patients are exposed to physical stress, such as surgery, dental procedures, or traumatic injury.^6)^ More than 25 mutations distributed across the FXIII-A and FXIII-B subunits have been reported as causative variants of this mild form of FXIIID. A previous review article^3)^ summarized reported missense mutations in *F13A1* and *F13B*; among cases involving homozygous or compound heterozygous mutations in *F13A1*, severe FXIII deficiency was reported in 68 cases, mild-to-severe deficiency in 1 case, and no cases were classified as mild. In contrast, among cases with heterozygous mutations alone, severe FXIII deficiency was reported in 2 cases, mild deficiency in 18 cases, and an undetermined phenotype in 1 case.

In this case, the FXIII activity increased to 59% 9 months after surgery, which may be explained by the resolution of chronic bleeding from gastric polyps and tumors following total gastrectomy. Nevertheless, this level remained below the normal range (≥70%), suggesting that the deficiency in this patient was unlikely to be explained solely by consumption. Genetic testing of *F13A1* and *F13B* revealed a heterozygous missense variant in *F13A1*, classified as a variant of uncertain significance. Furthermore, the heterozygous missense variant identified in the present case was not included among the missense variants associated with mild FXIIID listed in the aforementioned review.^3)^ This missense variant was not found in population databases, such as gnomAD v4.1.0 or ToMMo 60KJPN. Functional prediction analysis using multiple *in silico* predictive algorithms (SIFT4G, Polyphen-2, AlphaMissense) assessed this as probably damaging. However, because no copy number variants or genomic structural alterations were detected in the tests performed in this case, it is possible that a true pathogenic variant may have been missed. Although the clinical presentation was mild FXIIID, the absence of pathogenic variants on genetic testing precluded a definitive determination of whether the etiology was congenital or acquired.

In patients with FXIIID, prophylactic administration of FXIII concentrate every 4–6 weeks is recommended to maintain plasma FXIII levels at 3%–6% of normal.^10)^ According to the guidelines of the European Society of Anaesthesiology,^11,12)^ perioperative administration of 30 IU/kg of FXIII concentrate is recommended in cases with FXIII activity <60% or in those showing bleeding tendencies. Our patient had a history of gastrointestinal bleeding and was scheduled to undergo major surgery; therefore, the FXIII concentrate was administered twice at a dose of 30 IU/kg—once before and once after surgery. This regimen effectively prevented perioperative bleeding. Previous reports have described successful hemostasis after the administration of cryoprecipitate and fresh frozen plasma for postoperative bleeding following pancreaticoduodenectomy,^13)^ as well as the use of FXIII concentrate to control postoperative hemorrhage after laparotomy for sigmoid volvulus.^14)^

Another important consideration in this case was the surgical strategy. Because the patient had FXIIID with a potential risk of perioperative bleeding, the extent of lymph node dissection was carefully considered. Although the tumor was staged as cT1b, D1 lymph node dissection was selected to reduce the risk of bleeding complications while maintaining oncological safety. In addition, robot-assisted surgery was chosen as a minimally invasive approach. Although the superiority of robotic surgery over laparoscopic or open approaches cannot be determined from a single case, the robotic platform provides 3D magnified visualization, tremor filtration, and articulated instruments that allow precise manipulation. These features may facilitate meticulous dissection and careful hemostatic control in patients with an increased risk of bleeding. To our knowledge, this is the first report to describe robot-assisted gastrectomy in a patient with FXIIID. This case highlights the importance of considering FXIIID in patients with an unexplained bleeding history despite normal routine coagulation tests. Careful perioperative management with FXIII replacement therapy enabled safe completion of robot-assisted total gastrectomy. Recognition of such coagulation disorders may help prevent unexpected perioperative bleeding during major gastrointestinal surgery. In addition, this case suggests that minimally invasive robotic surgery may be a feasible surgical option when meticulous dissection and precise hemostatic control are required in patients with rare coagulation disorders.

Our patient had a family history of gastric polyposis and was also found to have multiple gastric polyps. Based on the diagnostic criteria,^15)^ she was preoperatively diagnosed with JPS and gastric cancer. Genetic testing revealed a pathogenic germline variant of *SMAD4*. A review that included 171 Japanese patients with JPS reported that gastric cancer occurred in 31 patients (18.1%).^16)^ There are no reports on the relationship between JPS and FXIIID; therefore, these 2 rare diseases are considered to occur incidentally. At present, there is no evidence suggesting a biological or genetic association between JPS and FXIIID in the literature. Therefore, these 2 rare diseases are considered to have occurred incidentally in this patient.

## CONCLUSIONS

We report a case of JPS-associated gastric cancer complicated by FXIIID successfully treated with robot-assisted total gastrectomy. Recognition of FXIIID and appropriate perioperative management may help prevent unexpected bleeding during major gastrointestinal surgery.

## DECLARATIONS

### Funding

The authors received no specific funding for this work.

### Authors’ contributions

T. Saito drafted the manuscript and contributed to the discussion.

T. Ishiguro performed the surgical procedure.

H. Ishida, Y. Mori, and N. Tanabe contributed to the discussion and genetic testing.

H. Ishikawa and N. Chiyonobu joined the surgical procedure.

K. Kiuchi, N. Irie, T. Ito, and Y. Kumagai contributed to the discussion.

All authors read and approved the final manuscript.

### Availability of data and materials

Data are available from the corresponding author upon reasonable request.

### Ethics approval and consent to participate

This study was conducted in accordance with the principles of the Declaration of Helsinki. This study was approved by the local ethics committee of Saitama Medical Center (No. 2099-IV).

### Consent for publication

Informed consent to publish has been obtained from the patient.

### Competing interests

The authors of this manuscript have no conflicts of interest to disclose.
